# Living and Learning Partnerships on Age-Friendly University Campuses

**DOI:** 10.1093/geroni/igaa057.1731

**Published:** 2020-12-16

**Authors:** Joann Montepare, Kimberly Farah

**Affiliations:** Lasell University, Newton, Massachusetts, United States

## Abstract

Campuses and communities have a long and varied history of partnerships around educational efforts. In response to current shifts in age demographics and the number of adults aged 65+ increasing dramatically, new partnerships around living and learning are emerging. In particular, university-based retirement communities (UBRCs) associated with institutions of higher education are growing as housing options for older adults in the United States. The establishment of the Age-friendly University (AFU) initiative offers a guiding framework for how UBRC partnerships may be effectively mounted. This presentation will use the case example of the Lasell University – Lasell Village partnership to demonstrate how AFU principles can be implemented through age-friendly curricular programming and intergenerational exchange. Logistical considerations such as individualized learning plans for UBRC residents, an on-campus location, shared resources, and governance policies will be also be discussed as components of successful partnerships.

